# Carcinome neuroendocrine du pancréas à propos d'un cas

**DOI:** 10.11604/pamj.2015.20.269.5489

**Published:** 2015-03-19

**Authors:** Pierlesky Elion Ossibi, Khalid El Haoudi, Salima Rezzouk, Khalid Ibn Majdoub, Imane Toughrai, Said Ait Laalim, Khalid Mazaz

**Affiliations:** 1 Service de Chirurgie Viscérale B, CHU Hassan II, Fès, Maroc

**Keywords:** carcinome, neuroendocrine, pancréas, carcinoma, neuroendocrin, pancreas

## Abstract

Les tumeurs neuroendocrines du pancréas sont des tumeurs rares avec une incidence estimées à 1/100000 habitants. Nous rapportons le cas d'un carcinome neuroendocrine bien différencié du pancréas chez un patient de 55 ans, diabétique sous traitement admis dans notre structure pour un syndrome tumoral de l'hypochondre droit. Le traitement a consisté en une duodéno-pancréatectomie céphalique.

## Introduction

Les tumeurs neuroendocrines se définissent par l'expression de protéines de structure et de produits de sécrétion hormonaux communs aux neurones et à l'ensemble des cellules endocrines [1, 2]. La plupart de ces tumeurs sécrètent des polypeptides responsables d'un syndrome clinique d'hypersécrétion hormonale qui permet de les détecter à un stade précoce. A l'inverse, un petit nombre de ces tumeurs (environ 15%) sont dites non fonctionnelles [3] car elles ne s'accompagnent d'aucune manifestation clinique d'hypersécrétion hormonale mais se révèlent plus tardivement par un syndrome tumoral locorégional ou par des métastases. Nous rapportons l'observation d'un patient de 55 ans, diabétique sous traitement opéré dans notre structure pour un syndrome tumoral de l'hypochondre droit avec découverte en post opératoire à l'examen anatomo-pathologique de la pièce d'un carcinome neuroendocrine bien différencié du pancréas.

## Patient et observation

**Figure 1:**
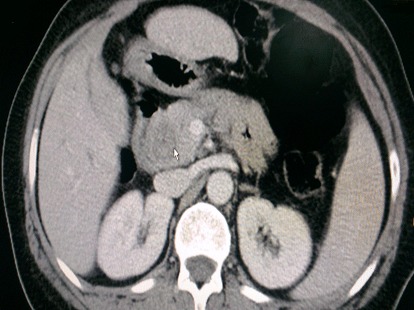
Image scannographique de la tumeur

Il s'agit d'un patient de 55 ans, diabétique sous anti diabétique oral depuis 3 ans, qui consulte dans notre structure pour des douleurs de l'hypochondre droit avec un ictère. L'histoire de la maladie remonte à environ 1 mois par la survenue des douleurs de l'hypochondre droit accompagnées d'un ictère d'allure cholestatique et des vomissements intermittents sans autres signes associés. L'examen clinique a trouvé un ictère cutanéo-muqueux franc, des lésions de grattage avec des douleurs à la palpation profonde de l'hypochondre droit. Sur le plan biologique, un syndrome de cholestase a été mis en évidence avec une hyperglycémie. La tomodensitométrie thoraco-abdomino-pelvienne a objectivé une masse tissulaire de la région ampullaire, avec extension ganglionnaire locorégionale. Lésion tissulaire de 35 mm au voisinage de la première anse jéjunale faisant évoquer en premier une adénopathie (Figure 1). La Bili-IRM a mis en évidence une grosse vésicule biliaire non lithiasique avec une importante dilatation de la voie biliaire principale à 20 mm et de voies biliaires intra hépatiques en rapport avec un processus lésionnel tissulaire du carrefour bilio-digestif de 17 mm de diamètre compatible avec un ampullome waterien. L’écho-endoscopique est revenue en faveur d'un processus tumoral ampullaire à développement intra canalaire responsable d'une dilatation des voies biliaires en amont. Le patient a bénéficié d'une duodéno-pancréatectomie céphalique. L'examen anatomie pathologique de la pièce de la doudeno-pancréatectomie céphalique est revenu en faveur d'un carcinome neuroendocrine bien différencié. Un bilan hormonal a été réalisé en post opératoire mais sans particularité. Le dossier a été discuté au colloque pluridisciplinaire et il a été décidé d'une surveillance.

## Discussion

Les tumeurs neuroendocrines du pancréas sont des tumeurs rares avec une incidence estimées à 1/100000 habitants [4]. La fréquence relative des tumeurs non fonctionnelles du pancréas endocrine est en nette diminution ces dernières années, en raison de la recherche systématique de polypeptides hormonaux, normalement sécrétés par le pancréas ou ectopiques, par méthode radio-immunologique ou immunohistochimique sur coupes tissulaires, voire par des méthodes plus modernes d'hybridation in situ. Leur proportion est passée de 41 à 15% entre 1950 et 1981 dans la littérature mondiale [5]. En l'absence de traduction clinique en rapport avec une sécrétion hormonale, ces tumeurs sont découvertes à un stade souvent très évolué à l'origine d'un syndrome tumoral associant un ictère cholestatique, douleurs abdominales, perte de poids et vomissements, soit, plus rarement, par la palpation d'une masse abdominale à l'examen clinique. Plus exceptionnellement, la manifestation clinique peut être inaugurée par une hémorragie digestive. C'est parfois encore l’évolution inhabituellement lente d'une tumeur initialement diagnostiquée comme étant un adénocarcinome qui amène à une relecture histologique et au diagnostic de tumeur neuroendocrine. Du fait de la multiplication des investigations radiologiques non invasives de l’étage abdominal, les tumeurs endocrines non fonctionnelles du pancréas peuvent être découvertes fortuitement lors d'une échographie ou d'un examen tomodensitométrique. La tumeur représente alors un véritable « incidentalome pancréatique » dont il conviendra de déterminer la nature.

L'examen tomodensitométrique (TDM) permet une bonne exploration de la région pancréatique. La taille de la tumeur, son siège dans le pancréas sont précisés. Il s'agit habituellement d'une tumeur solide. Lorsqu'elle est de volume important, elle peut être remaniée par des phénomènes de nécrose localisée lui donnant un aspect inhomogène, kystique. Les clichés après injection vasculaire permettent de mettre en évidence le caractère hypervasculaire de la lésion. L'extension aux éléments de voisinage, la présence d'adénopathies volumineuses ou de métastases hépatiques permettent d'emblée d'affirmer la malignité. L'imagerie par résonance magnétique (IRM) procure des renseignements morphologiques comparables au scanner et permet d’évoquer le diagnostic de TPE grâce au signal hypo-intense en T1 et au signal hyperintense en T2 de la tumeur [6]. La cytoponction guidée sous échographie ou TDM permet d'affirmer la nature endocrinienne de la tumeur pancréatique. Cette précision est importante en présence de tumeurs volumineuses, présentant une extension per-pancréatique (vasculaire) ou métastatique qui constituerait une contre-indication à l'exérèse chirurgicale en cas de tumeur exocrine. Le dosage plasmatique des hormones pancréatiques n'a pas d'indication en l'absence de signe d'appel, à l'exception du dosage du polypeptide pancréatique dont la sécrétion exclusive n'entraîne habituellement pas de traduction clinique. Le traitement des tumeurs endocrines est multidisciplinaire. Les tumeurs endocrines peu différencié ont un très mauvais pronostic et sont traité par la chimiothérapie systémique. La chirurgie d'exérèse est le seul traitement curatif des tumeurs endocrines bien différencié. Elle a comme objectif: prolonger la survie en réséquant la tumeur primitive et ses éventuelles métastases ganglionnaires et / ou hépatiques; contrôler un éventuel syndrome hormonal; prévenir ou traiter les complications locales [7].

Le traitement des tumeurs non fonctionnelles pancréatiques endocrines est chirurgical, permettant d'obtenir la guérison des formes bénignes et des rémissions prolongées en cas de formes malignes et métastatiques. Lorsque la tumeur est de petit volume (diamètre inférieur à 5 cm), et limitée au pancréas, l'absence de certitude quant à sa bénignité impose une résection réglée: duodéno-pancréatectomie céphalique avec conservation pylorique en cas de siège céphalique, pancréatectomie caudale ou corporéo-caudale lorsqu'elle siège sur le corps ou la queue. La conservation de la rate est techniquement réalisable et sera discutée en fonction des impératifs carcinologiques éventuels. En cas de tumeurs occupant la totalité du pancréas, la spléno-pancréatectomie totale associée à un curage ganglionnaire régional (c'liaque, hépatique, mésentérique supérieur) est indiquée. Elle reste licite pour ce type de tumeur, même en cas d'envahissement veineux splénique ou portal et sera complétée par une résection cunéiforme ou segmentaire de la veine porte, selon l’étendue du thrombus néoplasique. En cas de métastase hépatique, lors de l'intervention, la réalisation simultanée ou différée de l'exérèse d'une métastase unique est indiquée. Le plus souvent il s'agit de métastases diffuses dans les deux lobes. L'exérèse pancréatique reste licite compte tenu de l'intensité du syndrome tumoral et de la possibilité d'une évolution prolongée. En cas de tumeur très volumineuse, de siège corporéo-caudal, on préférera, à la spléno-pancréatectomie totale, une spléno-pancréatectomie subtotale poussée qui permettra de conserver l'intégrité du cadre duodénal et la voix biliaire principale avec un résidu de pancréas non tumoral. Le traitement médical de ces tumeurs a été proposé, soit comme complément à l'exérèse chirurgicale de tumeurs malignes, soit comme traitement isolé en présence de tumeurs jugées non résécables [8]. La chimiothérapie est essentiellement représentée par la streptozotocine isolément ou en association avec la doxorubicine ou le 5-fluoro-uracile. L'administration d'interféron a été proposée après échec de la chimiothérapie, ou en association avec la chimiothérapie. L'utilisation de la somatostatine a été plus récemment proposée. En dépit de taux de réponse variant de 10 à 50%, l'efficacité de ces traitements médicaux n'a pas été statistiquement démontrée mais des cas de survies paradoxales ont été rapportés [9]. Le suivi après traitement est adapté au type histologique de la tumeur endocrine. Alors que les carcinomes peu différenciés nécessitent une surveillance rapprochée tous les 2 à 3 mois pour évaluer la réponse à la chimiothérapie, les tumeurs endocrines bien différenciés requièrent un suivi espacé (tumeurs à temps de doublement long, plusieurs années étant souvent nécessaires pour mettre en évidence une progression lente).

## Conclusion

Les tumeurs neuroendocrines du pancréas sont rares. Leur diagnostic est encore vraisemblablement sous-estimé. L'imagerie joue un rôle primordial dans la localisation de la tumeur primitive, en permettant de faire un bilan d'extension local et à distance, d'assurer le suivi après traitement, de rechercher un syndrome de prédisposition aux tumeurs et de rechercher un second cancer associé.

## References

[1] Solcia E, Capella C, Kloppel G, Rosai J, Sobin LH (1997). Tumors of the endocrine pancreas. Tumors of the pancreas.

[2] Heymann MF, Moreau A, Chetritt J, Murat A, Leborgne J, Le Neel JC (1996). Pathologic study with immunohistochemistry of 61 pancreatic endocrine tumors in 16 patients suffering from multiple endocrine neoplasia type I (MEN I): Review of the literature. Ann Pathol..

[3] Vinik AI, Moattari AR (1989). Treatment of endocrine tumors of the pancreas. Endocrinol Metab Clin North Am..

[4] Thomas Walter, Julien Forestier, Catherine Lombard-Bohas (2010). Carcinome endocrine bien différencié pancréatique métastatique: discussion de la stratégie thérapeutique. Cancéro dig.

[5] Azoulay D, Sauvanet A, Bonnichon PH, Louvel A, Chapuis Y (1990). Tumeurs « non fonctionnelles » du pancréas endocrine: Concept, diagnostic et traitement. J Chir..

[6] Carriere F, Legmann P, Hazebrouck V, Abecassis JP, Pariente D, Bonnin A (1992). Place de l'IRM dans le diagnostic des tumeurs endocrines du pancréas. J Radiol..

[7] Kianmanesh R, O'Toole D, Sauvanet A, Ruszniewski P, Belghiti J (2005). Traitement chirurgical des tumeurs gastro-entéro-pancréatique. J Chir..

[8] Eriksson B, Skogseid B, Lundquist G, Wide L, Wilander E, Oberg K (1990). Medical treatment and long term survival in a prospective study of 84 patients with endocrine pancreatic tumors. Cancer..

[9] Eckhauser FE, Cheung PS, Vinik AI, Strodel WE, Lloyd RV, Thomson NW (1986). Non functioning malignant neuroendocrine tumors of the pancreas. Surgery..

